# Actinomycosis of the Nasal Septum: A Rare Entity

**DOI:** 10.7759/cureus.19475

**Published:** 2021-11-11

**Authors:** Catarina Lombo, Carlos Matos, Fausto Fernandes

**Affiliations:** 1 Otolaryngology - Head and Neck Surgery, Hospital Senhora da Oliveira de Guimarães, Guimarães, PRT; 2 Otolaryngology - Head and Neck Surgery, Hospital da Luz Guimarães, Guimarães, PRT

**Keywords:** sinonasal mass, nasal obstruction, obstructive sleep apnea, actinomyces, nasal septum, actinomycosis

## Abstract

Actinomycosis is a rare bacterial infection that can affect almost any site in the body. Its occurrence at the nasal septum is extremely rare. We present the case of an 84-year-old diabetic woman, with a past medical history of breast cancer that came to medical attention because of progressive nasal obstruction and severe sleep apnea. Nasal endoscopy revealed a submucosal mass at the anterior nasal septum obstructing both nasal cavities and extending to the anterior hard palate. With a cancer metastasis in mind, she was submitted to surgical resection of the lesion through a Rouge-Denker approach, with the final histologic diagnosis of actinomycosis. She was then treated with a three-month cycle of amoxicillin and remains without recurrence. This case describes a rare disease that should be considered in the differential diagnosis of sinonasal lesions, especially in diabetic patients and after dental procedures.

## Introduction

*Actinomyces* are Gram-positive non-acid fast filamentous anerobic (or microaerophilic) bacteria that belong to the normal flora of the oral cavity, gastrointestinal (GI), and urogenital tracts [1]. In rare circumstances, *Actinomyces* can become pathogenic after mucosal injury and cause a subacute or chronic granulomatous infection known as actinomycosis, which can mimic a large array of pathologies from malignancies to tuberculous infections [1-2]. The evolution of the infection varies from insidious, painless, and slowly progressive to fast dissemination and painful course [3-4]. This disease can be classified accordingly to the involved anatomic site into cervicofacial, thoracic, abdominopelvic, central nervous system, musculoskeletal or disseminated [2, 5]. Although up to 60% of infections occur at the cervicofacial region [6], the involvement of nasal cavities is remarkably rare, with few cases described in the literature [3, 5, 7-11]. Consequently, the clinical features and treatment protocol are not established yet.

We report the case of a nasal actinomycosis that caused the destruction of septal and hard palate cartilaginous-bony structures.

## Case presentation

An 84-year-old Caucasian woman was referred to the otorhinolaryngology clinic due to a three-month history of severe sleep apnea and bilateral permanent nasal obstruction. Additionally, she reported mucous rhinorrhea, without epistaxis. She denied smell disturbance, headache, nasal pain, pruritus, or sneezing. There were no systemic symptoms such as fever, weight loss, or night sweats. She denied any history of previous trauma, surgery, or dental procedures.

Her past medical history included a breast carcinoma with bone dissemination, for which she received immunotherapy. There was also a history of controlled hypertension, type 2 diabetes, and dyslipidemia. She had no smoking history or alcohol consumption.

**Figure 1:**
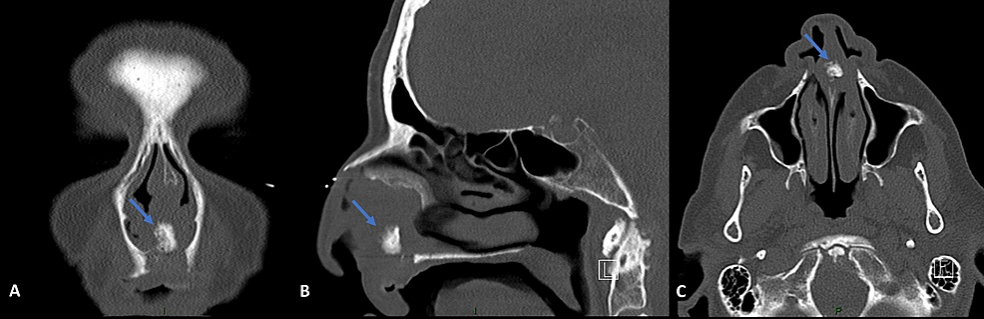
Preoperative paranasal sinuses CT scan. Paranasal sinuses CT scan showing an intraseptal submucosal mass with a central calcification (arrow) in coronal (A), sagittal (B), and axial (C) views.

Nasal endoscopy demonstrated a submucosal tender septal mass obstructing both nasal cavities. There were neither crusts nor purulent discharge and mucosa was intact. In the oral cavity, there was a tender absence of a portion of the bone in the hard palate. Teeth were fixed and free of caries. The CT scan showed a hypodense mass with an inside calcification destroying the anterior septum and hard palate (Figure 1). 

Considering the clinical presentation and patient’s medical history, the first diagnostic hypothesis was a metastasis from previous breast cancer or another type of malignancy. However, the tender consistency and unremarkable mucosal appearance also raised the hypothesis of a malignant granuloma of midline or granulomatous systemic disease (granulomatosis with polyangiitis or sarcoidosis).

An incisional biopsy and MRI were suggested to the patient, which she declined. With a malignancy in mind, however, she agreed with surgical excision of the lesion. 

A Rouge-Denker approach was performed, with submucosal dissection of the neoplasm, which had a soft consistency and granulomatous yellowish appearance. The anterior septal quadrangular cartilage was destroyed by the mass and was removed with part of the bony septum and nasal crest. The septal and hard palate mucosa were preserved. There were no surgical complications and the patient improved symptoms afterward.

**Figure 2:**
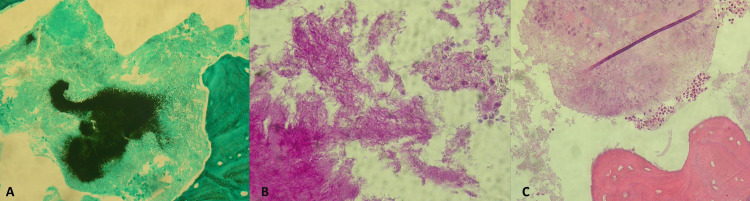
Surgical specimen histopathology findings. Histopathology analysis of surgical specimen showing colonies of *Actinomyces*. A, Grocott staining; B, Periodic Acid-Schiff staining; C, Hematoxylin-eosin staining

However, the histological result was unpredictable: there was no evidence of malignant transformation, but colonies of Actinomyces were detected instead (Figure 2).

**Figure 3:**
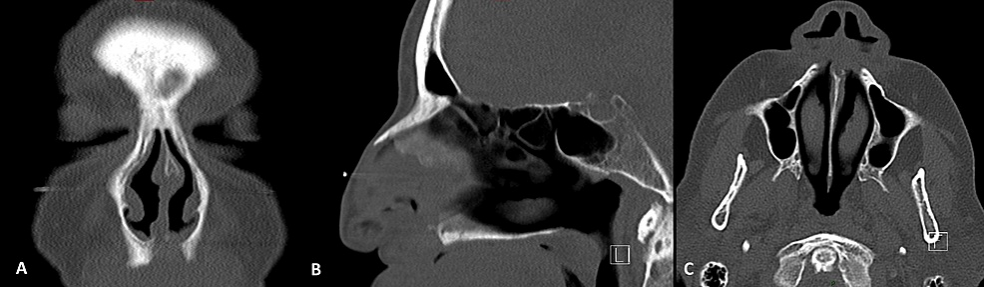
Postoperative paranasal sinuses CT scan. Absence of the calcified lesion at post-operative paranasal sinuses CT scan after six months of follow-up in coronal (A), sagittal (B), and axial (C) views.

Then, due to the multiple comorbidities, the patient was prescribed long-term amoxicillin 500 mg twice a day for three months. The postoperative CT scan, performed six months after surgery, was unremarkable (Figure 3) and she is currently alive after eight months of follow-up, without neither symptoms nor signs of disease recurrence. 

## Discussion

*Actinomyces* is a commensal microorganism of the oral, GI, and genital mucosa. However, sometimes it can become pathogenic, usually following the destruction of normal mucosa in the presence of other bacteria that predispose to an anerobic environment, enabling the growth of these colonies. Therefore, actinomycosis most often occurs after surgical interventions such as dental extraction procedures, abdominal surgery, or intrauterine dispositive insertion. An immunocompromised state and systemic diseases like diabetes are also known risk factors for this disease, which is the case of the present patient [1-3].

The infection usually courses with the development of granulation tissue, necrosis, abscesses, and sulfur granules capable of destroying surrounding structures [11]. Symptoms are similar to chronic rhinosinusitis caused by other microorganisms, such as purulent discharge [10]. In contrast, in the present case, the symptoms were mainly obstructive. Image findings usually consist of a mass with focal bone destruction and central calcifications, which can also occur in fungal infections such as aspergillomas or neoplasms [6-7, 9, 12-13].

The diagnosis may be difficult through regular cultures considering Actinomyces are fastidious microorganisms and its isolation may take up to 14 days, with the risk of being considered negative in the normal laboratory setting. Newer laboratory techniques such as matrix-assisted laser desorption ionization-time of flight mass spectrometry can help identify this bacteria when cultures are negative [14]. However, most of the time, the diagnosis is suspected through histological analysis, with the detection of sulfur granules and colonies of *Actinomyces* [15].

In the present case, the submucosal invasion of septal and hard palate structures caused sleep apnea and nasal obstruction, and the past medical history of the patient induced the suspicion of neoplasia. Fortunately, the final diagnosis had a better prognosis. The cause of *Actinomyces* inoculation in the submucosa is unknown. Probably the tissue structural changes promoted by hyperglycemia seen in diabetic patients increased the susceptibility to infection. As far as we know, there are only three reports in the literature of actinomycosis located in the nasal septum [5, 8, 10].

Owing to the rarity of this disease, there is no established treatment protocol. It usually consists of surgical debridement followed by long course antibiotics. Although penicillin G is usually the drug of choice, the antibiotics used have been multiple and can be tailored to disease extension, severity, and location [2, 5-7, 9-10, 12]. In our case, due to the age and multiple comorbidities of the patient, a simple course of amoxicillin was preferred and the patient improved and remains free of recurrence after eight months of follow-up.

## Conclusions

Actinomycosis of nasal cavities is a rare presentation of the disease and mimics multiple pathologies such as neoplasms, mucoceles, and fungal infections. It should be considered in cases of nasosinusal lesions with bone destruction, especially in immunosuppressed patients and in those who have been subjected to any kind of surgical procedure.

## References

[1] Stabrowski T, Chuard C (2019). [Actinomycosis]. Rev Med Suisse.

[2] Boyanova L, Kolarov R, Mateva L, Markovska R, Mitov I (2015). Actinomycosis: a frequently forgotten disease. Future Microbiol.

[3] Vorasubin N, Wu AW, Day C, Suh JD (2013). Invasive sinonasal actinomycosis: case report and literature review. Laryngoscope.

[4] Karanfilian KM, Valentin MN, Kapila R, Bhate C, Fatahzadeh M, Micali G, Schwartz RA (2020). Cervicofacial actinomycosis. Int J Dermatol.

[5] Park KS, Lee DH, Lim SC (2021). Actinomycosis of the nasal cavity. Braz J Otorhinolaryngol.

[6] Kim SD, Kim DS, Choi KU, Cho KS (2018). Nasal cavity actinomycosis mimicking rhinolith. J Craniofac Surg.

[7] Ozcan C, Talas D, Görür K, Aydin O, Yildiz A (2005). Actinomycosis of the middle turbinate: an unusual cause of nasal obstruction. Eur Arch Otorhinolaryngol.

[8] Kingdom TT, Tami TA (1994). Actinomycosis of the nasal septum in a patient infected with the human immunodeficiency virus. Otolaryngol Head Neck Surg.

[9] Woo HJ, Bae CH, Song SY, Choi YS, Kim YD (2008). Actinomycosis of the paranasal sinus. Otolaryngol Head Neck Surg.

[10] Lee DH, Yoon TM, Lee JK, Lim SC (2020). Nasal septum actinomycosis mimicking mucocele. J Craniofac Surg.

[11] Könönen E, Wade WG (2015). Actinomyces and related organisms in human infections. Clin Microbiol Rev.

[12] Zalagh M, Akhaddar A, Benariba F (2012). Chronic rhinorrhea revealing an actinomycotic rhinolithiasis with ectopic tooth. Int J Oral Maxillofac Surg.

[13] Vrinceanu D, Dumitru M, Patrascu OM, Costache A, Papacocea T, Cergan R (2021). Current diagnosis and treatment of rhinosinusal aspergilloma (Review). Exp Ther Med.

[14] Jeican II, Barbu Tudoran L, Florea A (2020). Chronic rhinosinusitis: MALDI-TOF mass spectrometry microbiological diagnosis and electron microscopy analysis; experience of the 2nd otorhinolaryngology clinic of Cluj-Napoca, Romania. J Clin Med.

[15] McHugh KE, Sturgis CD, Procop GW, Rhoads DD (2017). The cytopathology of Actinomyces, Nocardia, and their mimickers. Diagn Cytopathol.

